# Reinterpreting I^2^ Thresholds: Toward Context‐Specific Heterogeneity Assessment in Evidence Synthesis

**DOI:** 10.1002/cesm.70091

**Published:** 2026-06-22

**Authors:** Arturo J. Martí‐Carvajal, David L. Streiner

**Affiliations:** ^1^ Cátedra Rectoral de Medicina Basada en la Evidencia Social Sciences Doctoral Program (Health and Society), Universidad de Carabobo Valencia Carabobo Venezuela; ^2^ Facultad de Medicina Universidad Francisco de Vitoria Madrid Spain; ^3^ Department of Psychiatry and Behavioural Neurosciences McMaster University Ontario Hamilton Canada

**Keywords:** evidence synthesis, heterogeneity, I‐squared, meta‐analysis, meta‐regression, patient‐important outcomes, systematic review methodology

## Abstract

**Background:**

The Cochrane Handbook's *I* ^2^ categorization system (0%–25% “low”, 25%–75% “moderate”, ≥ 50% “high” heterogeneity) defines the standard approach to interpreting heterogeneity in meta‐analysis and informs thousands of systematic reviews each year. Despite its widespread use, its logical coherence and its role as an analytical decision tool have received limited formal examination.

**Objective:**

To examine whether the Cochrane Handbook's I^2^ categorization system includes overlapping category definitions that create ambiguous classification, and to propose context‐specific frameworks that preserve I^2^ as a decision tool for heterogeneity exploration.

**Methods:**

We conducted a structured genealogical analysis of key methodological sources related to the Cochrane Handbook's *I* ^2^ categorization. We examined whether individual *I* ^2^ values can satisfy more than one category. We analysed the categorization using principles from formal logic, philosophy of science, and statistical theory. We traced the development of I^2^ interpretation from its original formulation to current Cochrane guidance. We developed context‐specific frameworks based on patient‐important outcome categories.

**Results:**

The *I* ^2^ categorization system includes overlapping definitions in which identical values satisfy more than one category (e.g., *I* ^2^ = 50% corresponds to both “moderate” [25%–75%] and “high” [≥ 50%]). This structure assigns single values to multiple categories and departs from principles that require consistent and mutually exclusive classification. The primary literature provides limited explicit theoretical or empirical justification for the selected thresholds. The current approach uses *I* ^2^ as an interpretive endpoint rather than as a decision tool for heterogeneity exploration. These features reduce interpretive clarity and obscure the role of clinical context in heterogeneity assessment.

**Conclusions:**

The current I^2^ categorization system introduces ambiguity and leads to inconsistent analytical decisions across outcome contexts. Evidence synthesis requires context‐specific frameworks in which interpretation reflects outcome type and expected variability. We propose the PIOHA framework to align heterogeneity assessment with clinical relevance while preserving I^2^ as an analytical decision tool.

**Clinical Relevance:**

Systematic reviews inform clinical guidelines and patient care. Context‐specific heterogeneity assessment supports analytical decisions that reflect the clinical importance and expected variability of patient‐important outcomes.

## Summary

- The Cochrane *I* ^2^ categorization system creates logical impossibilities where single values simultaneously satisfy contradictory categories (e.g., *I* ^2^ = 50% is both “moderate” and “high”).
- Systematic literature review reveals no theoretical or empirical justification for these categories in primary sources from 2002 to 2024, representing “orphaned methodology”.
- The categorical system violates elementary statistical principles including mutual exclusivity, information preservation, and context sensitivity that would be unacceptable in introductory coursework.
- Current approach misuses I^2^ as interpretive endpoint rather than analytical decision tool for meta‐regression and heterogeneity exploration in studies with ≥ 10 trials.
- Context‐specific frameworks are proposed where I^2^ = 20% in all‐cause mortality requires different analytical decisions than I^2^ = 20% in quality‐of‐life outcomes.
- Patient‐Important Outcome Heterogeneity Assessment (PIOHA) framework recommended to align heterogeneity interpretation with clinical relevance rather than arbitrary statistical thresholds.
- This analysis challenges fundamental assumptions about methodological certainty in evidence synthesis and calls for individualized I^2^ interpretation strategies.

## 1 Introduction

Meta‐analysis represents the methodological cornerstone of evidence‐based medicine, with systematic reviews providing the highest level of evidence in clinical decision‐making hierarchies. The interpretation of between‐study heterogeneity constitutes one of the most consequential methodological decisions in evidence synthesis, directly influencing clinical recommendations, guideline development, and regulatory decisions [1].

The *I* ^2^ statistic, introduced by Higgins and Thompson [2], quantifies the proportion of total variation attributable to heterogeneity rather than sampling error. This metric has achieved universal adoption, appearing in virtually all contemporary meta‐analyses and serving as the primary tool for heterogeneity assessment in systematic reviews. Critically, *I* ^2^ was designed as an analytical decision tool to guide meta‐regression and heterogeneity exploration, particularly in meta‐analyses with ten or more studies.

However, the Cochrane Handbook's categorical interpretation framework has transformed I^2^ from an analytical decision tool into a categorical interpretation endpoint (0%–25% “low”, 25%–75% “moderate”, and ≥ 50% “high” heterogeneity) [3]. Two issues motivated this analysis. First, the categorical labels “low”, “moderate”, and “high” lack an explicit rationale that links their boundaries to clinical context, outcome type, and expected variability. Second, the category definitions overlap, so that a single *I* ^2^ value can be assigned to more than one interpretive category. The concern is therefore not categorization itself, but the absence of a clear rationale and of mutually exclusive boundaries. The widespread use of rule‐of‐thumb thresholds for interpreting *I* ^2^ has also been questioned in recent methodological research. Wang et al. showed that reliance on a single point estimate of *I* ^2^ may be misleading due to variability across estimators and inherent uncertainty [4]. However, this limitation concerns the estimation of *I* ^2^ and is distinct from the present analysis.

This study focuses on the interpretation of *I* ^2^ categories rather than on limitations in estimation, complementing prior work on estimator variability [4]. Current categorical approaches may also obscure the context‐dependent nature of heterogeneity assessment.

A systematic reviewer confronting *I* ^2^ = 20% in all‐cause mortality faces different analytical considerations than one encountering *I* ^2^ = 20% in quality‐of‐life outcomes, yet current frameworks provide identical categorical labels for these clinically distinct scenarios.

This investigation examines these issues and proposes context‐specific analytical frameworks that preserve the use of *I* ^2^ as a decision tool for heterogeneity exploration. The goal is not to abandon *I* ^2^ but to support its use within an analytical framework that reflects clinical context. The widespread use of *I* ^2^ categorization in systematic reviews and guidelines highlights the importance of ensuring that its interpretation remains methodologically coherent and clinically meaningful.

### 1.1 Logical Structure of Categorical Interpretation

The categorical interpretation of *I* ^2^ assigns values to predefined ranges such as “low”, “moderate”, and “high” heterogeneity [3]. At boundary values, a single *I* ^2^ estimate may fall within more than one category. For example, an *I* ^2^ value of 50% may be described as both “moderate” (25%–75%) and “high” (≥ 50%) under commonly used thresholds. This feature of the categorization introduces ambiguity in interpretation, as the same value may be associated with more than one label. Such cases illustrate how fixed categorical ranges applied to a continuous measure may lead to overlapping classifications [5].

### 1.2 Boundary Values and Classification Overlap

The overlap is not limited to a single example. Similar boundary issues can occur at other points within the categorization system. An *I* ^2^ value of 25% may be associated with both “low” and “moderate” heterogeneity, while values of 50% and 75% may be associated with both “moderate” and “high” heterogeneity.

These examples show that the issue is structural rather than dependent on a particular meta‐analysis. The concern lies in the way the categories are defined, not in the statistical estimation of *I* ^2^ itself.

### 1.3 Logical and Statistical Considerations

Classification systems are most useful when their categories are clear, mutually exclusive, and linked to an explicit rationale. The epistemological tradition of cognitive systematization has long emphasized that coherent interpretive structures require connectedness, consistency, and rational integration among their components [6]. Formal epistemology has shown that classification systems based on overlapping or incompatible interpretive principles may generate ambiguity and reduce interpretive coherence [7]. For a continuous statistic such as *I* ^2^, interpretation also needs to preserve information and remain sensitive to clinical and methodological context.

The current categorical approach can be difficult to apply in this way because overlapping ranges may reduce interpretive clarity. A context‐specific approach seeks to preserve the continuous nature of *I* ^2^ while using it as a signal for further analytical consideration.

Interpretation of heterogeneity is not limited to I^2^. Complementary measures such as τ^2^ quantify between‐study variance, while prediction intervals describe the range within which effects of future studies may lie. These measures address different aspects of heterogeneity and support interpretation that extends beyond a single summary statistic.

Current methodological guidance discourages reliance on fixed thresholds and emphasizes interpretation that integrates statistical measures with clinical context. Within this perspective, I^2^ contributes as one component of a broader analytical framework rather than as a standalone basis for interpretation.

The proposed context‐specific approach aligns with this development by interpreting I^2^ in relation to outcome type, expected variability, and clinical context, while remaining compatible with complementary measures of heterogeneity.

### 1.4 Genealogical Analysis of *I* ^2^ Categorization

We conducted a structured genealogical analysis of key methodological sources related to the development and interpretation of the *I* ^2^ statistic. This analysis included the original description of *I* ^2^ by Higgins and Thompson [2], successive editions of the Cochrane Handbook [3, 8], and selected methodological literature on heterogeneity interpretation. This approach is interpretive and focuses on the evolution of methodological guidance rather than on exhaustive identification of all published sources.

Higgins and Thompson introduced *I* ^2^ as a measure for quantifying the proportion of variability attributable to between‐study heterogeneity rather than sampling error. The original paper focused on the mathematical and analytical properties of the statistic, including its role in guiding exploration of heterogeneity.

The categorical interpretation later appeared in Cochrane guidance and became widely used in evidence synthesis. The reviewed sources did not identify a detailed theoretical or empirical rationale for the specific threshold values or qualitative labels. This observation supports the need to distinguish the statistical value of I^2^ from the interpretive framework imposed on it.

### 1.5 I^2^ as Analytical Decision Tool, the Categorical Transformation, and Context‐Dependent Analytical Decisions

The original conception of *I* ^2^ emphasized its role as an analytical decision tool, particularly for determining when further exploration of heterogeneity may be warranted in meta‐analyses with multiple studies. Higher *I* ^2^ values may signal the potential utility of investigating sources of variation through subgroup analysis or meta‐regression, whereas lower values may support the use of pooled estimates within an appropriate analytical context.

The subsequent use of categorical interpretation has shifted this role toward a fixed interpretive endpoint. This shift may reduce flexibility by replacing context‐sensitive analytical reasoning with predefined labels. As a result, similar categorical descriptions may be applied to outcomes that differ in clinical importance and expected variability. The use of such labels may also limit further exploration of heterogeneity by placing emphasis on classification rather than analysis.

Interpretation of *I* ^2^ may therefore be informed by outcome type and clinical context. The same *I* ^2^ value can be associated with different analytical considerations across outcomes. For example, an *I* ^2^ value of 20% in all‐cause mortality may prompt examination of population characteristics, healthcare system factors, or methodological differences, given the expectation of relative biological consistency. In quality‐of‐life outcomes, an *I* ^2^ value of similar magnitude may reflect variability related to measurement properties, cultural context, and differences in patient‐reported experience. For functional outcomes, an *I* ^2^ value of 20% may be considered in relation to variation in intervention delivery, assessment timing, and population characteristics.

### 1.6 A Context‐Specific Framework: The Patient‐Important Outcome Heterogeneity Assessment (PIOHA)

We propose the PIOHA framework to support context‐sensitive interpretation of between‐study heterogeneity. It extends the emphasis on patient‐important outcomes to the analytical use of the *I* ^2^ statistic. PIOHA does not rely on universal categorical thresholds. It treats *I* ^2^ as an analytical signal whose interpretation depends on outcome type, expected variability, and clinical context. The examples below illustrate how identical I^2^ values can be associated with different analytical considerations across outcome domains. The framework does not introduce outcome‐specific thresholds; it provides a structure for context‐informed interpretation that requires empirical evaluation.

### 1.7 Mortality and Major Morbidity

High biological and clinical consistency across studies characterizes this domain. I^2^ may warrant examination of population characteristics, healthcare system factors, or methodological differences. Heterogeneity assessment can focus on differences in care delivery, baseline risk, or study design.

#### 1.7.1 Quality‐of‐Life and Patient‐Reported Outcomes

Subjective experience and dependence on cultural, linguistic, and contextual factors define this domain. Variation across studies is expected and does not necessarily indicate methodological inconsistency. Interpretation of I^2^ reflects variability related to measurement properties, cultural influences, and implementation factors. It can distinguish contextual variation from artefacts related to measurement or reporting.

#### 1.7.2 Functional and Behavioural Outcomes

Dependence on intervention delivery, adherence, and healthcare context defines this domain. Variation across settings is expected. Interpretation of I^2^ relates to differences in implementation, healthcare settings, and population characteristics. Analysis can identify patterns that inform generalisability and implementation strategies.

### 1.8 Conceptual Implications

PIOHA repositions I^2^ as a decision‐guiding signal rather than a categorical endpoint. The framework avoids universal labels such as “low”, “moderate”, or “high” and supports interpretation that reflects outcome‐specific expectations and clinical context.

This approach preserves the continuous nature of *I* ^2^ and supports analytical decisions such as subgroup analysis, meta‐regression, or cautious interpretation of pooled estimates without reliance on fixed categories.

### 1.9 Advantages of Context‐Specific Frameworks

Context‐specific frameworks align heterogeneity assessment with the clinical expectations associated with different outcome types. Analytical decisions are therefore grounded in the anticipated patterns of variation rather than in universal categories.

This approach preserves the analytical function of the *I* ^2^ statistic as a decision‐guiding signal, rather than reducing it to a categorical endpoint. It supports the use of *I* ^2^ to inform further investigation, including subgroup analysis and meta‐regression, in a manner that reflects outcome‐specific considerations.

By avoiding universal thresholds, context‐specific interpretation reduces internal contradictions inherent to overlapping categorical systems. It maintains analytical coherence while preserving the continuous nature of *I* ^2^ and its relevance for evidence synthesis.

### 1.10 Conceptual Considerations in Heterogeneity Interpretation

Interpretation of heterogeneity in evidence synthesis can be examined through established perspectives in the philosophy of science. From the perspective of Thomas Kuhn [9], variation across studies may reflect differences in underlying theoretical, methodological, and clinical contexts. Meta‐analyses often combine evidence generated under diverse assumptions and settings, and observed heterogeneity may therefore correspond to this diversity rather than to methodological variation alone. A context‐sensitive approach allows these differences to be interpreted in relation to outcome type and expected patterns of variation.

From the perspective of Karl Popper [10], interpretation frameworks benefit from clarity that allows analytical distinctions to be evaluated. When categorical labels are applied to continuous measures, interpretation may become less clearly defined. For example, a label such as “moderate heterogeneity” may correspond to a range of values that overlap with other categories. Approaches that relate I^2^ to outcome type and analytical context support clearer interpretation and more precise analytical reasoning.

From a semantic perspective, as discussed by Frederick Suppe [11], the interpretation of scientific measures involves relating quantitative expressions to their empirical and conceptual meaning. The use of universal categorical labels may not fully reflect this relationship when applied to a continuous statistic such as I^2^. Context‐specific frameworks support a closer connection between statistical measures and the clinical meaning of outcomes by incorporating contextual and outcome‐specific considerations into their interpretation.

### 1.11 Information Loss in the Categorization of Continuous Variables

Beyond logical contradictions, the *I* ^2^ categorization is not aligned with established statistical principles regarding continuous variables [12]. The theory of measurement distinguishes continuous from categorical scales and cautions against arbitrary transformation of quantitative data. This concern has long been recognized in methodological literature [13]. Dichotomization can reduce effect sizes by up to 60% and eliminate important relationships [14]. It also results in substantial loss of information and statistical power [15].

This approach is not easily reconciled with elements of Cochrane guidance. While the Handbook appropriately discourages dichotomization of continuous outcomes in primary studies, it simultaneously promotes categorization of the continuous *I* ^2^ statistic. Context‐specific frameworks preserve the continuous nature of I^2^ while providing clinically relevant analytical guidance.

### 1.12 Alignment With Cochrane Methodological Principles

**Figure 1:**
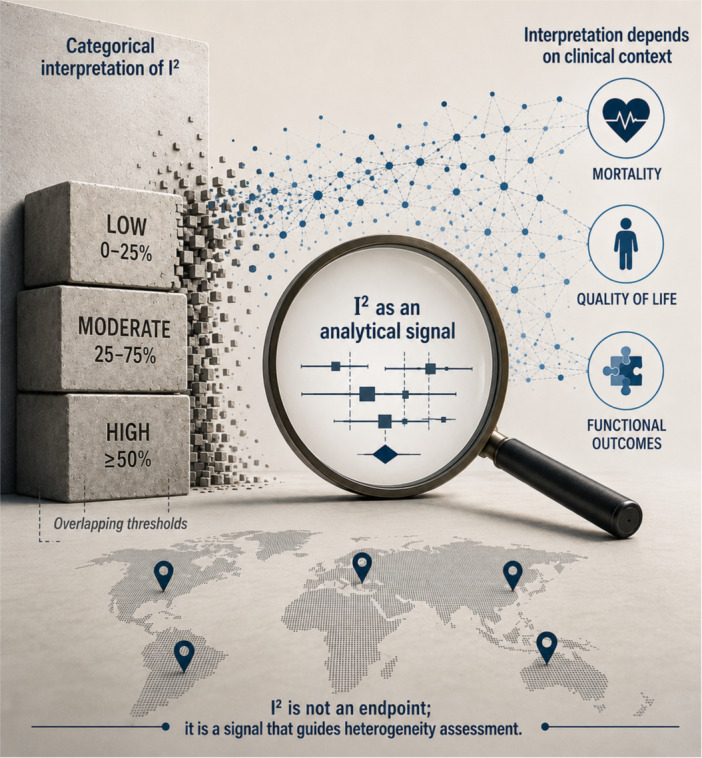
Conceptual representation of I^2^ interpretation in evidence synthesis. The left panel illustrates the conventional categorical interpretation of *I* ^2^ as fixed labels (“low”, “moderate”, “high”) with overlapping thresholds. The central panel presents *I* ^2^ as an analytical signal that guides heterogeneity assessment. The right panel illustrates context‐dependent interpretation according to outcome type, including mortality, quality of life, and functional outcomes. This conceptual framework underpins the proposed Patient‐Important Outcome Heterogeneity Assessment (PIOHA) approach.

The categorical interpretation of *I* ^2^ is difficult to reconcile with principles articulated within Cochrane methodology. Cochrane guidance discourages the categorization of continuous variables, emphasizes the need to interpret findings within their clinical context, and requires explicit methodological justification for analytical decisions [8]. It also expects internal logical consistency across methodological frameworks and prioritizes patient‐important outcomes in evidence synthesis. The use of universal, overlapping I^2^ categories conflicts with these principles, as it assigns fixed labels to a continuous measure and obscures context‐dependent interpretation. In contrast, context‐specific frameworks preserve the continuous nature of I^2^ and support heterogeneity assessment that reflects clinical relevance and outcome importance. Cochrane guidance also emphasizes the importance of interpreting heterogeneity in relation to clinical context and cautions against reliance on fixed thresholds. The present analysis builds on this perspective by examining the implications of overlapping categorical definitions and by proposing an approach that supports context‐informed interpretation. (see Figure 1).

## 2 Practical Consequences for Evidence Synthesis

Consider three meta‐analyses reporting the same *I* ^2^ value of 20%. In a mortality outcome, this value may raise questions about population characteristics, baseline risk, or study design, given the expectation of biological consistency. In a quality‐of‐life outcome, it may reflect expected variation related to measurement properties and contextual factors. In a functional outcome, it may be examined in relation to implementation, adherence, and healthcare setting.

Under a categorical approach, all three scenarios may receive the same label, despite differences in interpretation and analytical implications. This dependence on context is not unique to heterogeneity assessment. A monthly income of one US dollar would be interpreted as low in virtually any ordinary economic setting, whereas an income of US$1000 may be interpreted differently according to the cost of living and purchasing power. Similarly, extreme *I* ^2^ values may provide relatively stable analytical signals, while intermediate or boundary values require interpretation in relation to outcome type, expected variability, and clinical context. A context‐specific approach links the same *I* ^2^ value to distinct analytical considerations and supports decisions such as whether to explore heterogeneity, interpret pooled estimates with caution, or examine sources of variation.

These considerations extend to several domains within evidence synthesis. In guideline development, heterogeneity assessment can align with outcome importance and expected patterns of variation. In regulatory contexts, interpretation may reflect differences in populations, settings, and implementation rather than reliance on universal categories. In clinical practice, this approach supports the interpretation of evidence that integrates statistical measures with contextual factors.

Context‐sensitive interpretation also informs training and communication in evidence synthesis by supporting analytical reasoning that integrates statistical measures with clinical context. At a broader level, interpretation of heterogeneity reflects how methodological guidance is applied in practice. Context‐specific approaches strengthen the link between statistical measures and clinical interpretation and support the use of I^2^ as a tool for further analysis rather than as a standalone label. These approaches inform systematic reviews, guideline development, and clinical decision‐making, while clarifying variation across populations and settings.

### 2.1 Implementation of Context‐Specific Frameworks

The application of context‐specific frameworks in evidence synthesis involves integrating outcome type and clinical context into the interpretation of heterogeneity. For systematic reviewers, this requires identification of the primary outcome category, including mortality and major morbidity, quality‐of‐life and patient‐reported outcomes, or functional and behavioural outcomes. Interpretation of *I* ^2^ is then informed by the expected patterns of variation associated with each outcome type. This approach may guide decisions regarding further investigation of heterogeneity, including the consideration of subgroup analysis or meta‐regression, as well as the framing of findings within their clinical context.

In guideline development, heterogeneity assessment may be aligned with the prioritisation of outcomes and their relevance for decision‐making. Interpretation of evidence can take into account expected variation across studies, with consideration of how this variation may influence the strength and applicability of recommendations. This perspective supports a relationship between heterogeneity assessment and the evaluation of outcome importance.

For journal editors and peer reviewers, a context‐sensitive approach may inform the evaluation of methodological coherence in evidence synthesis. This includes consideration of whether heterogeneity is interpreted in relation to outcome type and clinical context, and whether analytical decisions are supported by a transparent rationale. Such an approach may contribute to the clarity and interpretability of published findings.

The integration of context‐specific frameworks also has implications for training and education in evidence synthesis. It supports the development of analytical reasoning that treats I^2^ as a decision‐guiding signal rather than as a categorical endpoint. Educational approaches may emphasise the role of clinical context, outcome classification, and structured interpretation of heterogeneity. The use of case‐based examples across different clinical domains may further support the application of these principles in practice.

## 3 Limitations and Considerations

Context‐specific frameworks support an approach to heterogeneity assessment that links statistical interpretation with clinical context and outcome type. This perspective contributes to methodological clarity by aligning analytical reasoning with the expected patterns of variation associated with different outcomes, while maintaining the use of *I* ^2^ as a quantitative measure within an appropriate interpretive framework.

The use of context‐sensitive interpretation may also inform the development of quality assessment approaches in evidence synthesis. Tools such as AMSTAR‐2 and ROBIS may incorporate consideration of whether heterogeneity assessment reflects outcome type and clinical context, and whether analytical decisions are supported by a transparent rationale.

This approach identifies several areas for further work within the evidence synthesis community. These include empirical evaluation of context‐specific frameworks, development of shared approaches to outcome‐informed heterogeneity assessment, and investigation of how such frameworks can be integrated into training, guideline development, and methodological guidance. Such efforts may support the consistent application of heterogeneity assessment that reflects both statistical measures and clinical interpretation. The genealogical analysis is not intended as an exhaustive systematic review of all methodological sources. It focuses on key texts that have shaped the interpretation of *I* ^2^ within evidence synthesis.

## 4 Conclusions

The categorical interpretation of *I* ^2^ assigns fixed labels to a continuous measure and may limit context‐dependent interpretation. The use of *I* ^2^ as an analytical decision tool can be supported through approaches that relate its interpretation to outcome type and clinical context.

The Patient‐Important Outcome Heterogeneity Assessment framework illustrates an approach in which heterogeneity assessment is informed by the expected patterns of variation associated with different outcomes. For example, an *I* ^2^ value of 20% may lead to different analytical considerations in all‐cause mortality and in quality‐of‐life outcomes.

This perspective supports heterogeneity assessment that integrates statistical measures with clinical interpretation. It aligns analytical reasoning with outcome importance and expected variability across studies.

The interpretation of heterogeneity remains central to evidence synthesis and its application in clinical and policy contexts. Approaches that preserve the continuous nature of *I* ^2^ while supporting context‐sensitive interpretation may contribute to clarity and consistency in analytical decision‐making.

## Author Contributions

**Arturo J. Martí‐Carvajal:** conceived the analysis, conducted genealogical research, developed context‐specific frameworks, drafted initial manuscript, critical revision, final approval. **David L. Streiner:** contributed statistical and methodological analysis, logical framework development, epistemological analysis, critical revision, and final approval. Both authors accept full responsibility for the integrity of the work and the accuracy of all claims presented.

## Funding

The authors have nothing to report.

## Conflicts of Interest

Arturo J. Martí‐Carvajal has been an editor of Cochrane systematic reviews since 2004 and has contributed to the development of Cochrane methodology guidelines. David L. Streiner has served on editorial boards of multiple journals publishing meta‐analyses and has contributed to statistical methodology development in clinical epidemiology. Both authors declare no competing interests.

## Supporting information

Supporting File

## Data Availability

Data sharing not applicable to this article as no datasets were generated or analysed during the current study. All logical arguments, literature searches, and analytical procedures are fully documented within the manuscript. The genealogical analysis can be independently verified through examination of cited Cochrane Handbook editions. No additional datasets are required to evaluate our claims.
